# Supplementary material for: Effect of Soybean Protein Isolate-7s on Delphinidin-3-O-Glucoside from Purple Corn Stability and Their Interactional Characterization
Source: Foods. 2022 Mar 22;11(7):895. doi: 10.3390/foods11070895 (PMC9254744; doi:10.3390/foods11070895)
Supplement: Supplementary file 1 [file foods-11-00895-s001.zip › foods-1630763-supplementary.pdf]

Figure S1: HPLC chromatograms of delphinidin-3-*O*-glucoside monomer

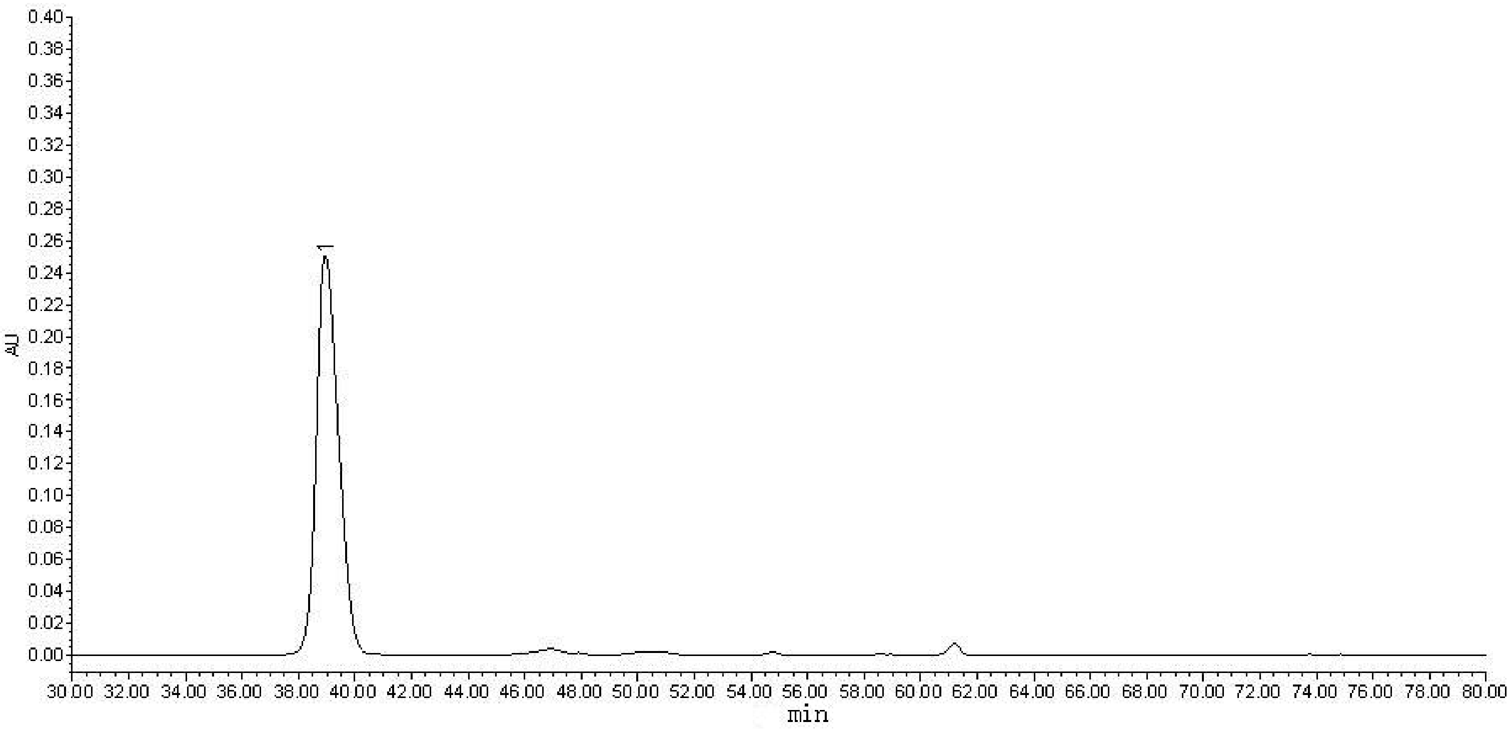

Table S1: Optimum design of soybean protein isolate-7s-delphinidin-3-*O*-glucoside stabilization system (pH 6.8) (mean ± SD) (n = 3)

No.	A: Complex proportion	B: Reaction temperature (°C)	C: Reaction time (min)	Abs‡
1	-1 (40:1)	-1 (50)	0 (60)	0.605
2	1 (30:1)	-1	0	0.677
3	-1	1 (70)	0	0.684
4	1	1	0	0.706
5	-1	0 (60)	-1 (30)	0.661
6	1	0	-1	0.693
7	-1	0	1 (90)	0.656
8	1	0	1	0.702
9	0 (20:1)	-1	-1	0.659
10	0	1	-1	0.701
11	0	-1	1	0.655
12	0	1	1	0.709
13	0	0	0	0.740
14	0	0	0	0.747
15	0	0	0	0.731
16	0	0	0	0.714
17	0	0	0	0.728

Source	Sum of square	df	Mean square	*F* value	*P* value	Significant
Model	0.033	9	0.003632	23.55	0.002	**
A	0.004465	1	0.004465	28.95	0.0010	**
B	0.005460	1	0.005460	35.40	0.0006	**
C	0.004805	1	0.004805	3.12	0.1209	
AB	0.005062	1	0.005062	3.28	0.1129	*
AC	0.009000	1	0.009000	0.058	0.8160	
BC	0.006760	1	0.006760	4.38	0.0746	
A^2	0.001220	1	0.001222	78.22	0.0001	**
B^2	0.004053	1	0.004053	26.28	0.0014	**
C^2	0.003019	1	0.003019	19.57	0.0031	**
Residual	0.001080	7	0.001542			
Lack of Fit	0.001008	3	0.003358	0.14	0.9327	Not significant
Pure Error	0.009788	4	0.002447			
Cor Total	0.034	16				
R^2_{adj}		0.9269				
R^2_{Pred}		0.9070				
C.V./%		1.73				

SPI-7s-D3G[†]: The complex of soybean protein isolate-7s and delphinidin-3-*O*-glucoside; pH 6.8: The system of pH 6.8; Abs[‡]: Absorbance; *: The difference was significant ($P < 0.05$); **: The difference was extremely significant ($P < 0.01$).
